# Modified Langendorff technique for mouse heart cannulation: Improved heart quality and decreased risk of ischemia

**DOI:** 10.1016/j.mex.2017.11.004

**Published:** 2017-11-14

**Authors:** Negar Motayagheni

**Affiliations:** Wake Forest Institute for Regenerative Medicine, 391 Technology Way, Medical Center Boulevard, Winston-Salem, NC 27157, USA

**Keywords:** Langendorff method, Arrhythmia, Isolated mouse heart, Isolated heart

## Abstract

Oscar Langendorff introduced the first method for isolating a heart with contractile activity in 1895. Since then, the Langendorff method has remained a powerful technique in cardiac research and has led to major advances in medicine. The primary goal of the Langendorff method is to provide an isolated heart with oxygen and metabolites via a cannula inserted into the aorta. The Langendorff heart is a complex in vitro technique used primarily in pharmacological and physiological research that allows the evaluation of multiple cardiac hemodynamic parameters including, but not limited to, contractility and heart rate. This article will first provide a brief background of the Langendorff method as well as details regarding organ isolation. Finally, the article will discuss the benefits of a new technique for hanging the isolated heart aorta and the benefits of this technique over traditional methods

## Graphical abstract

**Figure:**
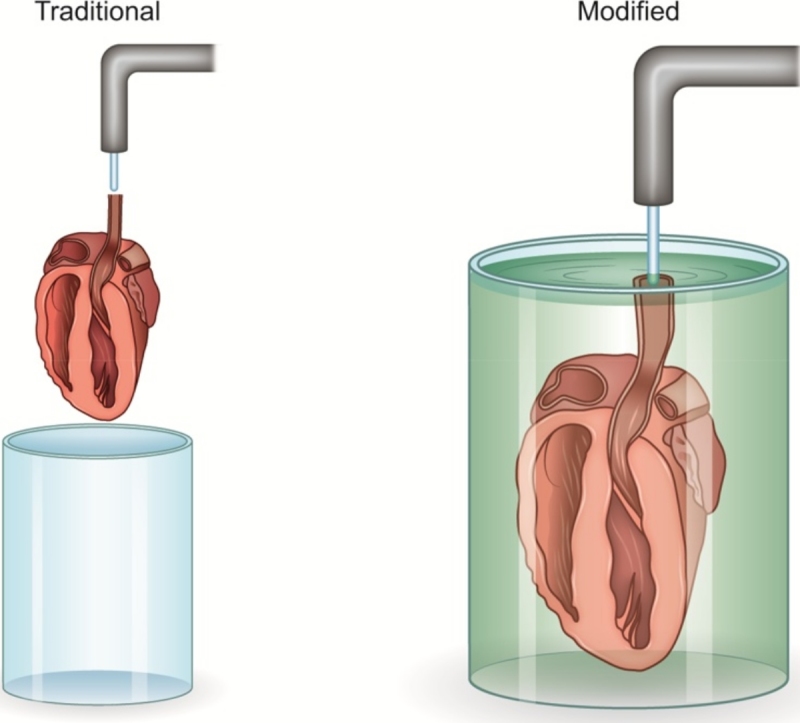

## Method details

In the Langendorff preparation, the heart is first surgically removed from the animal's body. Subsequently, the aorta is suspended in the Langendorff apparatus and the heart is perfused via the aorta, usually with a nutrient rich oxygenated solution [Krebs and Henseleit solution (KHB)]. The delivery of nutrients and oxygen allows the heart to continue beating and also allows the evaluation of the effects of different drugs on the heart [1], [2]. The Langendorff heart preparation relies on the constant pressure of the perfusion fluid to supply the myocardium with adequate oxygen and nutrients that may have been depleted during removal of the heart from the donor animal. The isolation of high-quality cardiomyocytes is critical for conducting successful experiments, and techniques for isolating murine heart cardiomyocytes are complex and time-sensitive. Major technical difficulties are related to the surgical procedures needed to cannulate the aorta to the Langendorff apparatus before starting the reperfusion. During this period, transient hypoxia and ischemia can cause damage to the heart, resulting in poor quality cells and affecting the results of the experiment [3]. The Langendorff method has led to many important advances in our understanding of ischemia–reperfusion injury, as well as cell based therapy and cardiac transplantation [4], [5], [6], [7], [8], [9], [10], [11], [12].

This article reviews the general principles of the Langendorff method and presents a novel modification of the Langendorff cannulation method that minimizes the effects of hypoxia and ischemia on experimental protocols. This method helps to produce high quality hearts for Langendorff experiments investigating cardiac parameters and ventricular myocytes.

## Principles of the Langendorff method

Most isolated perfused heart preparations are based on the method originally described by Langendorff [13]. Once the heart is removed from an anesthetized animal it is clipped to a cannula on the perfusion apparatus. The cannula is attached to the outflow of a reservoir containing an oxygenated perfusion solution. The majority of research protocols use a physiologic salt solution that mimics the content of plasma (KHB) that is delivered at 37 °C with physiological pH of 7.4 [14], [15], [16]. In each Langendorff experiment, a wide range of physiological, morphological, and pharmacological parameters, including contractile function, heart rate, cardiac metabolism, and electrical activity of the heart, can be evaluated. Another advantage of this technique is that the intact organ will spontaneously beat when place in an environment with the proper oxygenation, perfusion fluid, and temperature, making this method reasonably physiological. Moreover the heart is free from the influence of other organs, the systemic circulation, and signals from the central and autonomic nervous systems [17]. The Langendorff technique allows for the induction of ischemia, arrhythmia, and hypoxia to various degrees, which makes it a unique tool for the study of pathological cardiac conditions. The isolated heart preparation is valuable in studying the mechanisms underlying arrhythmias as well as ischemia-reperfusion. The model is also a valuable tool for studying the direct effect of drugs on the heart including cardiotoxicity. Additionally, a wide range of measurements can be done with the isolated heart to evaluate the mechanisms of acute cardiotoxicity [18]. Importantly, it should be noted that the potential disadvantages of the Langendorff isolated perfused heart technique are highly dependent on the skills of the investigator. The heart is vulnerable to contusion injuries, and there is a significant possibility of ischemic damage to the heart during its isolation and instrumentation. Improper preparation has been reported to have a great impact on the initiation and maintenance of ischemia-induced ventricular fibrillation in isolated rabbit hearts, making them less suitable for study [19]. Among different animal species, the isolated mouse heart preparation is more complicated because of its high heart rate and small size. The guinea pig heart, having extensive collaterals in the coronary circulation, is not suitable for ischemia studies [15], [20].

## Details of the Langendorff method

### Anesthesia and excision of the heart

Anesthesia can be induced either by the inhalation of volatile agents or injection [14], [15]. Efforts should be made to keep the animal free from stressful stimuli. In some protocols, heparin is intravenously injected prior to excision to prevent the formation of thrombi in the excised heart [21]. Following the onset of general anesthesia, the animal is placed in the supine position. To expose the thoracic cavity and the heart, the diaphragm is cut using a trans-abdominal incision. Immediately after excision, the heart is transferred into an ice-cold KHB solution in order to rinse the blood, temporarily stop the heart from beating, and to protect the heart from ischemic injury. The time of ischemia during this period should be minimized and kept constant, since a longer temporary ischemia period can induce ischemic preconditioning of the heart [14], [15].

### Cannulation of the aorta and re-establishment of vascular perfusion

It is important to inspect the cannula and tubes connecting the aortic cannula prior to cannulation to ensure that they are free of air bubbles. With any technique, laceration of the aorta should be avoided. The heart is then connected to the Langendorff apparatus with a clip, and the contractile function of the heart returns within seconds. It should be noted that there is a strong relationship between cardiac function and temperature [14], [15]. The temperature of the heart may be influenced by the temperature of the air surrounding the heart as well as the temperature of the perfusate [15]. Considering the importance of temperature effects, efforts should be made to maintain a correct and constant temperature. In some protocols, the heart is immersed in a solution of physiological saline at 37 °C to better control its temperature during the ischemic period. Suspending the heart from its aortic cannula in room air is a less desirable method for ischemia studies [19], [22].

### Preparation of the mouse heart

As noted above, the isolated mouse heart preparation is more complicated and challenging because of its rapid heart rate and small size [15]. Before anesthesia, the mouse is injected with heparin to reduce the risk of thrombus formation. There are several protocols for anesthesia based on different institutional guidelines. Following the induction of anesthesia, the mouse is placed in a supine position. A thoracotomy is performed to expose the heart.. The vessels are excised, and the heart is immediately transferred to ice-cold KHB solution. The mouse heart is handled gently to avoid a contusion injury to the mouse heart. In contrast with larger animals, the mouse heart needs to be trimmed for a better access to the aortic lumen . The aorta is held with forceps to expose the lumen, and the heart is rapidly transferred to the cannula [14], [15], [23].

An alternative method of mouse aortic cannulation is to place a dish containing ice-cold KHB under a microscope with the tip of the cannula connected to a syringe and under magnification the aorta is cannulated. The cannulated heart is then detached from the syringe and connected to the Langendorff apparatus [23]. Using a mouse heart in the Langendorff apparatus is challenging.

### Modified Langendorff apparatus

Since longer ischemic periods can lead to ischemic preconditioning of the heart, the time of ischemia during the set-up should be minimized and kept constant. A new technique has been devised that is cost effective, faster, cardioprotective, and does not require the use of a magnifying instrument. In the traditional technique, the mouse heart is exposed to the open air without protective solution during the challenging hanging process or is under the magnifying microscope. A simple modification of the Langendorff apparatus allows the mouse heart to be protected inside the perfusate and allows the heart to be hung within a few seconds. In this modification, the hanging needle is positioned inside the perfusate, allowing the heart to be kept under the surface of the perfusate while the needle is located close to the aorta in the perfusate. The aortic orifice is completely distended by matching the diameter of the needle with that of the aorta. The needle and aorta are clipped together, and the needle is separated from the syringe and reconnected to the Langendorff apparatus. This short-length needle can be either included in the main apparatus or kept as a readily available separate instrument.

### Disadvantages of the traditional technique

There are several disadvantages associated with the traditional Langendorff technique. The longer hanging time with this technique in the mouse heart leads to ischemic damage to the heart. Additionally, exposure of the heart to the air without a protective solution contributes to ischemia, tissue temperature changes, and inconsistent results. Because of the small size of the mouse heart, there is increased damage to the aorta and surrounding tissues, resulting in an increased failure rate and higher costs. Finally, the traditional technique requires an assistant or the use of a magnifying instrument.

### Advantageous of the new technique

This new technique provides improved visualization of the aortic orifice because of the absence of air pressure within the solution. There is decreased hanging time, and the heart is kept in a protective solution instead of being exposed to the air, providing a consistent temperature (Cold Kerb buffer kept in 4 c) and surrounding environment. Because the procedure is performed within the solution, the introduction of air bubbles is prevented. Additionally, both hands are free to work and no assistant or magnification is needed. Ultimately, this new technique causes less damage to the surrounding tissues with lower failure rates and costs. Most importantly, there is improved heart quality with less ischemic damage.

## Conclusion

The Langendorff isolated perfused heart has allowed many fundamental discoveries in cardiac physiology, pathology, and pharmacology over the past 100 years and is still one of the most powerful experimental designs in cardiovascular research and cardiovascular pharmacology. With the advent of genetically engineered mice, the isolated mouse heart, with its wide spectrum of research applications, has become a powerful technique for medical research. The Langendorff experimental method is commonly used by pharmacologists and toxicologists. Researchers face multiple challenges in the cannulation process of Langendorff heart that may significantly affects their protocols. The novel technique described above preserves heart quality and is ideal for use in cardiovascular research.
